# Combined Triboelectric and Piezoelectric Effect in ZnO/PVDF Hybrid-Based Fiber-Structured Nanogenerator with PDMS:Carbon Black Electrodes

**DOI:** 10.3390/polym14204414

**Published:** 2022-10-19

**Authors:** Vikas Narayan Thakur, Jeong In Han

**Affiliations:** Department of Chemical and Biochemical Engineering, Dongguk University-Seoul, Seoul 04620, Korea

**Keywords:** fiber nanogenerator, wearable computing device, ZnO, piezoelectric, PVDF, PDMS

## Abstract

We report a fiber-structured hybrid nanogenerator wearable device fabricated on a single polyethylene terephthalate (PET) textile cylindrical substrate. The device can be described as a capacitor with inner and outer carbon-black-dispersed poly dimethyl siloxane (PDMS:Carbon black) electrodes, and zinc oxide and polyvinylidene fluoride (PVDF) as the dielectric medium between the electrodes. The compositional analysis in terms of X-ray diffraction, Fourier transform infrared spectroscopy, and scanning electron microscopy of the synthesized ZnO/PVDF has been measured and analyzed. The combined effect of triboelectricity between PDMS:Carbon black and PVDF, and piezoelectricity in a ZnO/PVDF hybrid, was investigated. Current–voltage characteristics were observed with varying load from 0–20 g, and resistance was observed to be decreased with load. Compared to earlier reports, there was a significant enhancement in voltage (≈5.1 V) and current (≈92.5 nA) at 10 g. Due to the introduction of interfacial polarization between PVDF and ZnO, the piezoelectric properties and pressure sensitivity of the hybrid ZnO/PVDF is enhanced. The hysterical behavior in the device’s response while measuring voltage and current with varying time shows the signature of the triboelectric effect between PVDF and ZnO, as well as PDMS:Carbon black and ZnO/PVDF layers. Reduction of triboelectric behavior was confirmed with increasing relaxation time. Because of the enhancement in piezoelectricity, fiber-structured nanogenerator (FNG) ZnO/PVDF proved to a potential candidate to be used for wearable computing devices, such as smart watches and sports bracelets.

## 1. Introduction

Human-based energy-harvesting systems have become of great interest with the broad spread of mobile electronics [1]. Energy sources for portable electronic devices have been designed to provide sufficient and necessary electricity for devices with low power consumption. Propagation of IoT technology means many objects in daily life or business can be controlled anywhere and anytime, forming a ubiquitous environment [2]. Wearable computing devices refers to electronics which are able to be incorporated into clothing, and have sustained rapid development. Electronic textiles (e-textiles or smart textiles) are being actively studied for fabricating all types of wearable devices [3].

Many research groups have developed wearable devices on two-dimensional substrates, such as glass or flexible polymer [4,5,6,7,8,9]. However, flat-type substrate has many obstacles for wearable-device applications [10,11,12]. Wearable devices will ultimately be woven into fabrics for clothes; hence, cylindrical shaped substrates, similar to textiles, are more advantageous for low-cost manufacturing [13,14]. Substrate surface roughness is essential when fabricating electronics, since electrical and mechanical properties can significantly deteriorate if cracks form between layers [13,15,16]. Traditional textiles, such as wool and cotton yarns, consisted of an assembly of thin threads. Therefore, a single fiber is required with smooth interfaces, enhancing operational features for wearable devices [13]. An external energy source is required within the clothes to commercialize and adapt wearable devices [17,18]. Therefore, self-powering systems, particularly piezoelectric devices, have been widely studied in conjunction with various nanoscale materials [19]. Piezoelectricity refers to energy harvesting from various external forces, such as heat, pressure, and electric fields, and includes ferroelectricity, pyroelectricity, and piezoelectricity [20,21,22,23,24,25].

Zinc oxide (ZnO) is a promising material for practical next-generation technological applications. Generally, ZnO has a wurtzite structure, which polarizes under an external force. It also forms a variety of nanostructures, such as nanorods, nanowires, nanobelts, and nanoforests, depending on the hydrothermal synthesis conditions [19,26,27]. Generally, the substrate is submerged in a nutrient solution, and ZnO nanoparticles are grown on metal electrodes. A redox reaction between zinc and other metal ions eradicates the metal layer without requiring a screen mask [28].

Thus, non-reactive polymeric electrodes are required to prepare intact electrodes after a chemical reaction. Ferroelectric polymers combined with ceramics maximize energy-harvesting performance, and are commonly called hybrid nanogenerators [28,29,30]. PVDF is the most widely employed ferroelectric polymer due to its vital beta factor, i.e., spontaneous electrical polarization aligned by an applied electric field and the protective ceramic layer [31,32,33].

ZnO/PVDF composite have been reported for sensing humidity [34], corrosion [35], pressure, and temperature [36,37]. The researchers have used various electrodes such as reduced grapheme oxide (rGO), indium tin oxide (ITO)-coated PET, Au, etc. [28,38,39]; however, PDMS:Carbon black has never been used in this ZnO/PVDF hybrid structure. Therefore, in this article, we present a fiber-structured nanogenerator (FNG) fabricated on a PET cylindrical substrate with fully covered electrodes made of PDMS:Carbon black for pressure-sensing wearable computing devices. The device comprises a capacitor with inside and outside electrodes of PDMS:Carbon black with a ZnO/PVDF piezoelectric layer. A novel electrode was constructed by dispersing conductive carbon black in polydimethylsiloxane, which provides the advantages of piezoelectricity, high elasticity, good adhesion, heat durability, and chemical inertness. Electrical performance, i.e., average output voltage and current, was significantly enhanced by combining ZnO and PVDF in the energy-harvesting layer. Negative differential capacitance was discovered by applying weights to the device. We validate that the proposed FNG provides a maximum voltage up to 10 times that reported previously due to the combination of piezo- and triboelectric properties in all the layers.

## 2. Experimental Section

**Figure 1:**
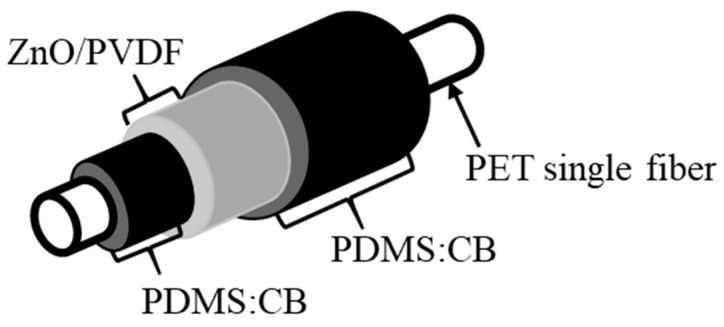
The labeled schematic of the fiber nanogenerator.

The proposed FNG consists of multiple thin films: a ZnO growth layer covered with PVDF, sandwiched between PDMS:Carbon black electrodes as shown in Figure 1. To linearize tangled PET fiber, 100 mm samples were held at 100 °C on a hot plate for 1 h, then rinsed with methanol, acetone, and deionized water at room temperature [15]. The polymer-based electrode was prepared by mixing PDMS (Dow Corning Sylgard 184) with its curing agent and 0.1 g carbon black powder (Alfa Aesar) in a 10:1:1 ratio in toluene solvent by magnetically stirring, because PDMS can be converted into electrically conductive material by adding 10% carbon black [40]. The composite, PDMS:Carbon black, was coated on the PET substrate by continuous dipping at a constant 5 mm/s, then placed in a desiccator to remove air bubbles, and cured at 65 °C for 30 min.

The seed solution of ZnO was prepared by mixing 1.1 g of zinc acetate dihydrate (Zn(CH_3_CO_2_)_2_·2H_2_O) in isopropyl alcohol (IPA) and sodium hydrate solution, while stirring drop-wise [41]. The nutrient solution was manufactured using the hydrothermal method by taking 1 M equimolar amount of hexamethylenetetramine ((CH_2_)_6_N_4_) and zinc nitrate hexahydrate (Zn(NO_3_)_2_·6H_2_O) in 200 mL deionized water and stirring for 24 h. Then, the solution was kept at 90 °C for 5 h [42].

The PDMS:Carbon-black-coated PET fiber was soaked in the seed solution and treated at 150 °C on a hot plate three times, then heated in the nutrient solution at 95 °C for 8 h [19,28]. The ZnO grown on the PDMS:Carbon-black-coated PET sample was dried under vacuum for approximately 24 h. The PVDF solution was prepared by mixing 1.5 g PVDF powder (Sigma Aldrich, Merck, Seoul, Republic of Korea) at a 6:4 volume ratio of acetone and Dimethylformamide (DMF) [27,28]. Then, the PET covered by PDMS:Carbon black and ZnO is coated with PVDF solution by a dipping method, and annealed at 65 °C (shown in Figure 1). The formation of ZnO, PDMS, and PVDF was confirmed by X-ray diffraction (XRD) using Ultima IV (Rigaku, Tokyo, Japan), Attenuated total reflectance (ATR)–Fourier transform infrared (FTIR) spectroscopy using a Nicolet 6700 (Thermo Fisher Scientific, Waltham, United States of America) and their surface morphology was studied using field-effect-scanning electron microscopy (FE-SEM) using a JSM-7100 (Jeol, Tokyo, Japan). After compositional analysis, electrical properties of the FNG—including electrode resistance, current-voltage (I–V) curves, average open-circuit and real-time output voltages, and short-circuit output current—were measured using a Keithley 6517 instrument (Tektronix, Beaverton, United States of America) and LabView (National Instruments, Texas, United States of America) while applying weights to the device.

## 3. Results and Discussion

### 3.1. Material Characterization

Figure 1 shows the various FNG layers on the cylindrical PET substrate covered by electrode layers and a ZnO/PVDF composite layer. The PET substrate has thermoelasticity, with a long rod shape similar to a textile strand. Although PET is generally insulative and requires specific equipment due to the cylindricity, it can be designed to be suitable for an electronic fiber by depositing a metal film or conductive polymer. Therefore, PDMS:Carbon black was uniformly coated on a PET substrate as an inner electrode and above a ZnO/PVDF layer as an outer electrode. The rubber-like property, due to cross-linked silicon groups in PDMS, maximizes electrode-film deformation while enhancing sensitivity to applied weights [40].

**Figure 2:**
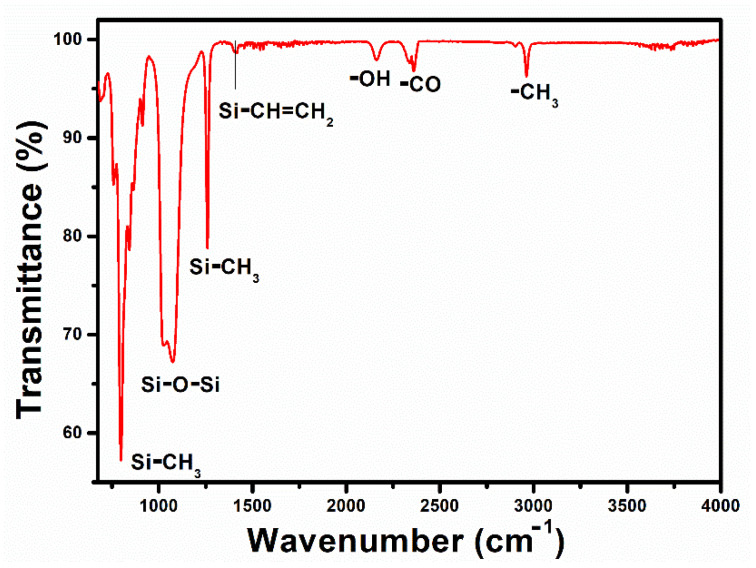
Fourier transform infrared spectroscopic pattern of PDMS.

ATR–FTIR was conducted to examine the existence of silicone links in synthesized PDMS, as seen in Figure 2. FTIR spectra indicated at the peaks of 790, 1072, 1259, 1408, 2164, 2362, and 2964 cm^−1^ (Si-(CH_3_)_2_, Si-O-Si, Si-CH_3_, Si-CH=CH_2_, -OH, -CO, and -CH_3_, respectively) [43,44]. Dispersing the conductive carbon black particles in the elastic PDMS produced a very flexible electrode through a simple coating process [40]. The piezoelectric layer, containing ZnO and PVDF, was evenly fabricated on the PDMS:Carbon black electrode by hydrothermal synthesis and dip coating. During the hydrothermal synthesis of zinc oxide, the inner electrode retained good adhesion with the substrate at 95 °C without causing an oxidation-redox reaction with zinc ions.

**Figure 3:**
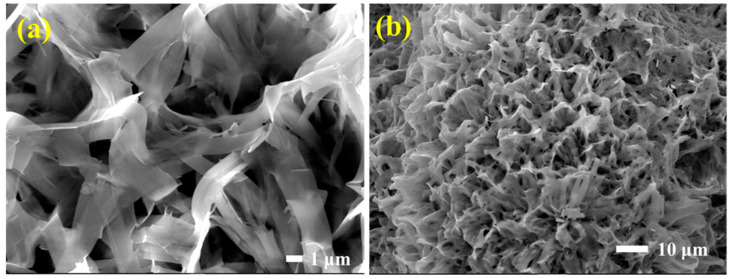
SEM images of (**a**) Hydrothermally synthesized ZnO and (**b**) ZnO/PVDF composite.

**Figure 4:**
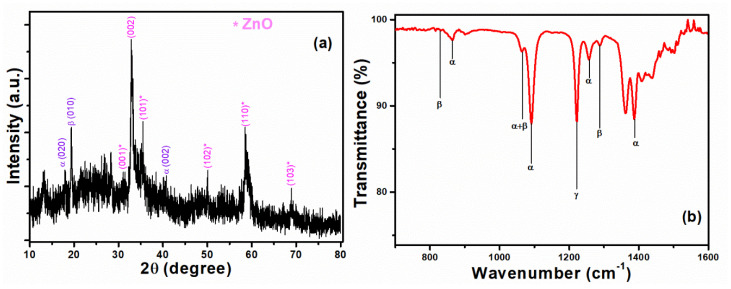
(**a**) XRD pattern of ZnO/PVDF composite, and (**b**) FTIR pattern labeled with α, β, and γ phases of PVDF.

Figure 3a,b shows SEM images of hydrothermally synthesized ZnO and ZnO/PVDF composite, respectively. Figure 3a shows ZnO alloyed as a tangled nanobelt (NB), and these nanobelts are agglomerated with micropores at the surface after PVDF coating, as shown in Figure 3b. The ZnO NB arrangement was affected by the PVDF powder being dissolved in DMF and acetone solvents during the immersion process [45]. DMF-treated ZnO/PVDF was characterized by the XRD method and the pattern is shown in Figure 4a. The diffraction planes of ZnO (marked with * in Figure 4a) are matched with JCPDS#36-1451 and for α, β, and γ phases of PVDF are JCPDS#42–1650, 38–1638, and 42–1649 [46].

PVDF porosity caused bonding shifts between ZnO NB composites, affecting the peaks in terms of their broadness and diffuseness. The effects on the peaks due to PVDF porosity confirmed that the composite structure had a broad shape, consistent with SEM images (Figure 3a). PVDF is a semi-crystalline polymer with α, β, and γ phases for different chain formations [47]. The PVDF β-phase has a permanent dipolar moment [48]; therefore, the β-phase has more polarity than the other phases of PVDF and, hence, it shows the ferroelectric and piezoelectric properties [49]. The PVDF β phase can be seen in the XRD pattern at 19.46°, and ATR-FTIR spectra show the PVDF β phase at 838, 1070, and 1288 cm^−1^ as seen in Figure 4b. Thus, PVDF enhanced piezoelectric performance through the β phase’s permanent polarization. Figure 4b shows 874, 1070, 1263, and 1383 cm^−1^ for the PVDF α phase and 1220 cm^−1^ for the PVDF γ phase [50,51,52,53,54].

### 3.2. Fiber Nanogenerator Electrical Properties

**Figure 5:**
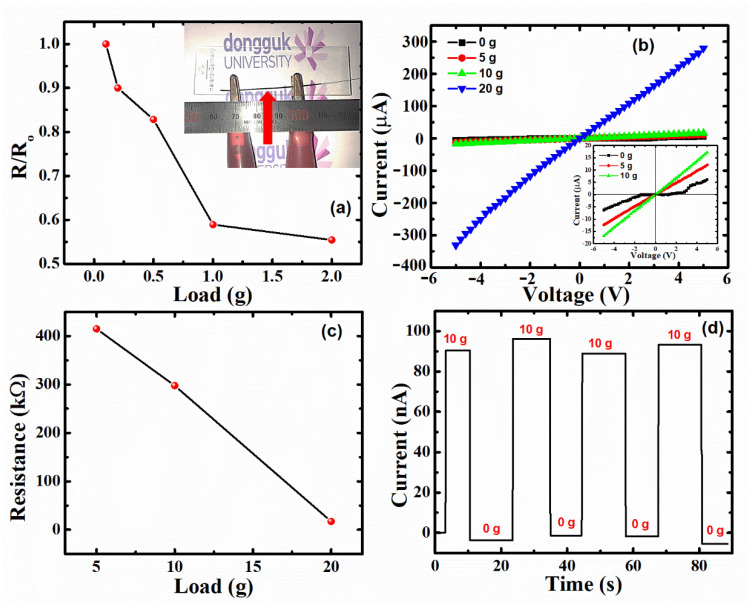
(**a**) The resistance variation while increasing the loading weight from 0.1 g to 2 g on PDMS:Carbon black electrode thin film as shown in the inset, (**b**) I–V curves with varying load from 0 to 20 g with zoomed-in inset figure of 0–10 g, (**c**) resistance calculated from the slopes in (**b**) with varying load from 5–20 g, and (**d**) the output current with 10 g load for ≈90 s under the short-circuit state.

PDMS:Carbon black was selected for the electrodes, and enhanced working performance due to its piezoelectricity [55,56] and large Young’s modulus [57]. Due to the piezoelectric nature of ZnO and PVDF, having the piezoelectric coefficients ≈ 6.08 and ≈2.24 pm/V [58], respectively, the device was constructed as a capacitor [1,31]. In Figure 5, the electrodes’ ability was assessed by measuring resistance variation. This was performed by compressing the weight ranging from 0.1 g to 2.0 g in the middle of the sample, which is pointed out at the inset of Figure 5a. This figure shows the variation of the ratio of resistance with load (*R*) to that of resistance without load (*R*_o_), i.e., *R*/*R*_o_, with applied load varying from 0.1 to 2 g. As load increases, the ZnO/PVDF NBs start coming close to each other, which tends to increase conductivity because of ohmic contact formation in the device.

Hybrid FNG piezoelectricity was estimated using a variety of electrical attributes such as I–V characteristics shown in Figure 5b, from −5 V to +5 V, with increasing load from 0 g to 20 g. There is a significant enhancement in current at 5 V from 17 to 280 µA. The inset of Figure 5b shows the zoomed-in image of Figure 5b for 0 g, 5 g, and 10 g. The inset shows that the I–V curve without load, i.e., 0 g, shows a non-linear resistive behavior similar to a diode, because the ZnO/PVDF NBs are sufficiently far from each other. However, with an increase in load towards 20 g, the I–V characteristics seem to have a linear resistive behavior similar to ohmic connections. The resistance values for 0 g, 5 g, and 10 g are calculated from the I–V curves and are shown in Figure 5c. This figure shows a linear decrease in resistance with an increase in load.

Figure 5c shows that resistance was 414.7, 297.4, and 17.0 kΩ at 5, 10, and 20 g loads, respectively, grounded on Ohm’s law. Since the deformation length is proportional to the external force from Hooke’s law and Young’s modulus [59], only the cross-sectional area should be affected, and therefore, resistance would tend to reduce proportionally. However, the resistance decreased by 117.3 Ω from the 5 to 10 g load, and 280.4 Ω from the 10 to 20 g load, corresponding to an extra 45.8 Ω reduction due to temporary triboelectric inductance caused by the capacitive structure and frictional contact between the dielectric materials, PDMS and PVDF. The response of the device was recorded in terms of current with time by applying and removing the load of 10 g for ≈90 s (Figure 5d) with an interval of 5–10 s. The figure shows a clear change in current from 0 g to 10 g, with stability during application or removal of load. The output current at 10 g is maintained in the range of 88.8–96.2 nA. This hysteresis behavior in the output current may also be due to the triboelectric effect in the device.

**Figure 6:**
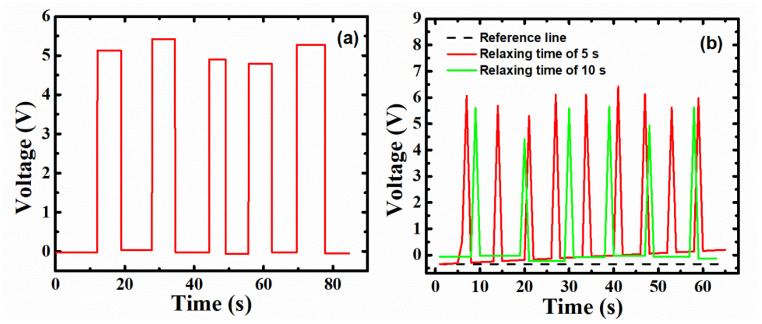
(**a**) Measured voltage for ≈90 s for loading and unloading 10 g, and (**b**) voltage is measured for ≈65 s with a relaxing period of ≈5 s and ≈10 s.

Nanogeneration capacity was also examined using output voltage with different measuring times for loading. Figure 6a shows the output voltage measured while loading and unloading (or relaxing) for ≈90 s. The measuring time while loading and relaxing kept an average period of 9 s, which also shows the stability in the measurement. The average voltage obtained for this duration is 5.1 V which is significantly higher than the maximum output voltage previously reported for devices fabricated from similar piezoelectric materials, i.e., ZnO/PVDF, as seen in Table 1. The maximum output voltage has been compared as per the mechanism, electrode material, and substrate. Table 1 depicts that the output voltage obtained from the piezoelectric effect is significantly lower than that of obtained from triboelectric effect. E.g., in ref. [60], output voltage is enhanced from 1 V to 4.5 V when comparing piezoelectric and triboelectric effects for a given electrode (Au) and substrate material (PVDF). Similarly, as depicted in Table 1, the output voltage obtained in the ZnO/PVDF reported in this article is significantly higher than that of reported literature, which is the signature of the triboelectric effect. In Figure 6b, two relaxing times have been taken of 5 s and 10 s, respectively, to check the triboelectric effect in the device. Under an open circuit, a 10 g load was applied and relieved for about 5 s and 10 s on the device, as shown in Figure 6b. The reference line is taken as per the initial voltage to see the gap between the output pulse relaxing line and the reference line.

The pulse output voltage was relatively high, lying near 5 V. However, as the number of input intervals increased, the voltage gap between the relaxing line and reference line values expanded, as shown in Figure 6b. The triboelectric charge was generated due to frictional contact of the dielectric materials with a brief relaxing time under 5 s, and the recovery time among piezoelectric materials differed [36]. Therefore, the case with a recovery time of 10 s appeared to be a consistent level compared to the time of the 5 s specimen, due to the reduced triboelectric effect shown in the case of a 10 s pulse.

**Figure 7:**
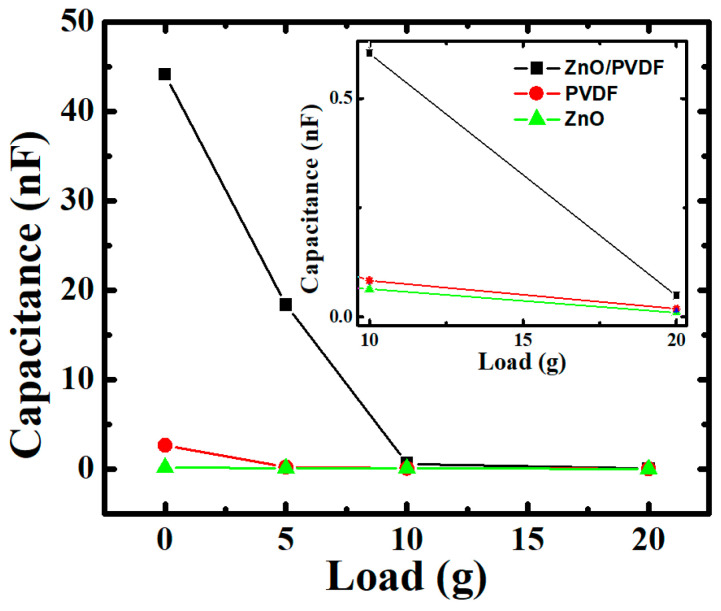
Comparison of capacitance change among three samples: ZnO, PVDF, and ZnO/PVDF with piezoelectric layers between PDMS:Carbon black electrodes, with varying load.

Figure 7 shows measured capacitance over a range of −5 to 5 V for three samples: ZnO, PVDF, and hybrid ZnO/PVDF with PDMS:Carbon black electrodes. Capacitance (C) is inversely proportional to the distance between the electrodes as given by Equation (1),

(1) $$C=\varepsilon \frac{A}{d},$$

where ε is the absolute permittivity of the medium, *A* is the electrode area, and *d* is the distance between the electrodes. For all three samples, capacitance decreases with load because of ohmic contact formation inside them with increasing load. Because of this, the dielectric constant is enhanced, due to the interfacial polarization among the polymer and fillers such as ZnO [65,66]. The capacitance was increased in hybrid ZnO/PVDF because of an increase in piezoelectric properties and absolute permittivity by combining two piezoelectric materials, i.e., ZnO and PVDF [65]. A magnified image of the capacitance at 10 g and 20 g is shown in the inset of Figure 7. Capacitance increased from ZnO (0.01 nF) and PVDF (0.018 nF) to hybrid ZnO/PVDF (0.048 nF) at 20 g.

However, the proposed hybrid FNG behaved somewhat non-typically due to the temporary triboelectric effects of adjacent materials as the load compressed the device. Although the distance between the charged media was decreased, the capacitance value was elevated. The results showed that the dielectric constant was also affected on the FNG, especially based on the fundamental working principle of triboelectricity, which acquires electrical charge from frictional contact between substances. Consequently, FNG performance was enhanced by combining piezoelectric materials in terms of energy provision, piezoelectricity, and triboelectricity.

## 4. Conclusions

In this article, a capacitor-structured FNG was synthesized and developed to take advantage of synergistic effects for energy-harvesting and piezoelectric materials. A detailed compositional analysis of ZnO/PVDF hybrid structure was analyzed. The proposed ZnO/PVDF hybrid sandwiched between PDMS:Carbon black electrodes inherited the core piezoelectric property, which enhanced performance under load. The detailed I–V characteristics were also investigated with varying loads from 0 g to 20 g, and stability was checked by applying and removing load alternately with time. The average electrical output was found to be ≈5 V under open-circuit, with an average short-circuit current of 92.5 nA at 10 g load, which is a significant enhancement compared to previously reported outcomes. This is because of the combination of piezo- and triboelectricity observed in the ZnO/PVDF which, due to such, can be used as an FNG. Similarly, the capacitance was also measured with varying load, and an increase in capacitance was observed from ZnO or PVDF to ZnO/PVDF due to an increase in piezoelectricity.

## Figures and Tables

**Table 1:** Comparison of output voltage produced in ZnO/PVDF hybrid flexible nanogenerator.

Electrode Material	Substrate Structure	Maximum Output Voltage	Mechanism	Reference
Au/Cr	Fiber-type	0.03 V	Piezoelectric	[28]
Ag	PVDF film	2 V	Piezoelectric	[39]
Ag	PVDF film	0.6 V	Piezoelectric	[38]
Ag	Cotton	0.09 V	Piezoelectric	[61]
Au	PVDF	1 V	Piezoelectric	[60]
Au	PVDF	4.5 V	Triboelectric	[60]
ITO/PET	PDMS	2.15 V	Triboelectric	[62]
ITO/PET	PDMS	3.5 V	Triboelectric	[63]
ITO/Al	PET	4.5 V	Triboelectric	[64]
PDMS:Carbon black	PET	5.1 V	Combined triboelectric and piezoelectric	This work

## Data Availability

The data presented in this study are available on request from the corresponding author.
